# Contributions of the left and right thalami to language: A meta-analytic approach

**DOI:** 10.1007/s00429-024-02795-3

**Published:** 2024-04-16

**Authors:** Talat Bulut, Peter Hagoort

**Affiliations:** 1 Max Planck Institute for Psycholinguistics, Nijmegen, The Netherlands; 2 Department of Speech and Language Therapy, School of Health Sciences, Istanbul Medipol University, Istanbul, Turkey; 3 Donders Institute for Brain, Cognition and Behaviour, Radboud University, Nijmegen, The Netherlands

**Keywords:** Thalamus, Language, Functional connectivity, Meta-analytic connectivity modeling, Brain networks

## Abstract

Background: Despite a pervasive cortico-centric view in cognitive neuroscience, subcortical structures including the thalamus have been shown to be increasingly involved in higher cognitive functions. Previous structural and functional imaging studies demonstrated cortico-thalamo-cortical loops which may support various cognitive functions including language. However, large-scale functional connectivity of the thalamus during language tasks has not been examined before. Methods: The present study employed meta-analytic connectivity modeling to identify language-related coactivation patterns of the left and right thalami. The left and right thalami were used as regions of interest to search the BrainMap functional database for neuroimaging experiments with healthy participants reporting language-related activations in each region of interest. Activation likelihood estimation analyses were then carried out on the foci extracted from the identified studies to estimate functional convergence for each thalamus. A functional decoding analysis based on the same database was conducted to characterize thalamic contributions to different language functions. Results: The results revealed bilateral frontotemporal and bilateral subcortical (basal ganglia) coactivation patterns for both the left and right thalami, and also right cerebellar coactivations for the left thalamus, during language processing. In light of previous empirical studies and theoretical frameworks, the present connectivity and functional decoding findings suggest that cortico-subcortical-cerebellar-cortical loops modulate and fine-tune information transfer within the bilateral frontotemporal cortices during language processing, especially during production and semantic operations, but also other language (e.g., syntax, phonology) and cognitive operations (e.g., attention, cognitive control). Conclusion: The current findings show that the language-relevant network extends beyond the classical left perisylvian cortices and spans bilateral cortical, bilateral subcortical (bilateral thalamus, bilateral basal ganglia) and right cerebellar regions.

**Supplementary Information:**

The online version contains supplementary material available at 10.1007/s00429-024-02795-3.

## Introduction

### Thalamus and language

Cortical-subcortical loops have been associated mainly with non-cognitive or lower cognitive functions such as sensorimotor and affective functions. This lack of association between cognition and subcortex could be due, at least partially, to a cortico-centric view or cortical bias pervasive in cognitive neuroscience literature. Potential contributors to this cortical bias include a priori hypotheses about language and other cognitive functions being mostly cortical, which guides region of interest definition; proliferation of surface-based imaging, recording and stimulation techniques such as magnetoencephalography, electroencephalography, functional near-infrared spectroscopy, and transcranial magnetic stimulation; relatively small size of subcortical nuclei and their high person-to-person variability; hemodynamic response function optimized for cortex not being sensitive enough to subcortical time course of activation; and cortex-based preprocessing and analytical procedures (e.g., coregistration, smoothing) not being ideal for subcortical nuclei (Janacsek et al. 2022; Llano 2013, 2016). Indeed, there is increasing evidence that subcortical structures contribute to cognitive functions such as working memory, cognitive control and language (Copland and Angwin 2019; Copland et al. 2021; Crosson 2021; Janacsek et al. 2022; Murphy et al. 2022; Shine et al. 2023; Theofanopoulou and Boeckx 2016; Ullman 2016).

**Fig. 1:**
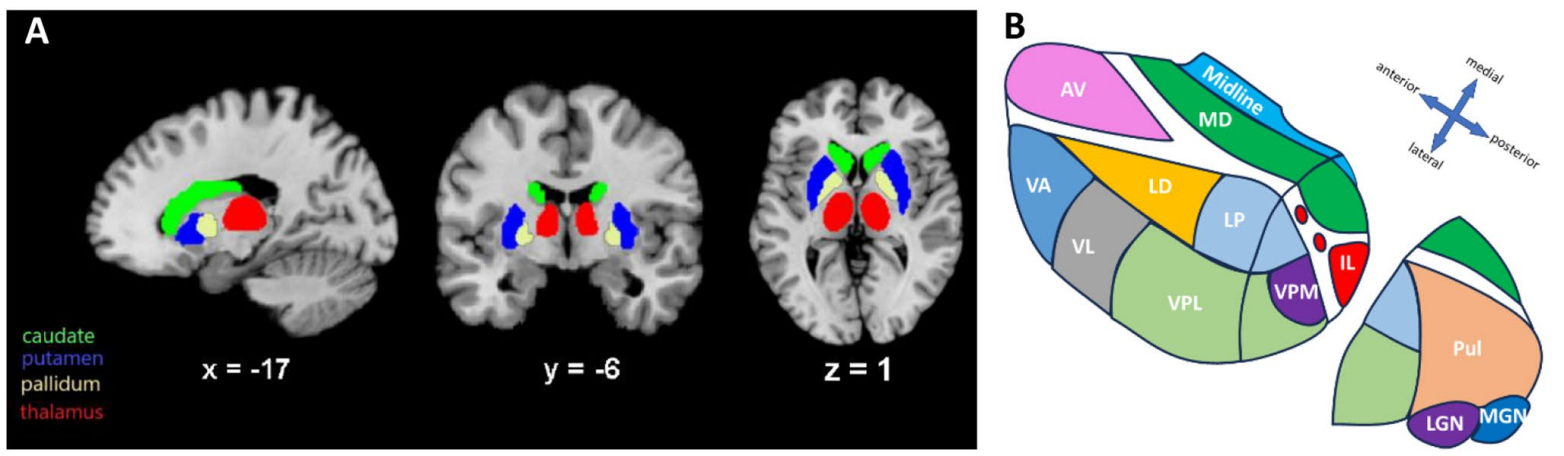
Parcellation of the thalamus and the basal ganglia in sagittal, coronal and axial planes based on the AAL3 atlas **(A)**; illustration of the left thalamic nuclei [AV: Anteroventral, LD: Lateral dorsal, LP: Lateral posterior, VA: Ventral anterior, VL: Ventral lateral, VPL: Ventral posterolateral, VPM: Ventral posteromedial, IL: Intralaminar, MD: Mediodorsal, Pul: Pulvinar, LGN: Lateral geniculate, MGN: Medial geniculate] **(B)**

One of the prominent subcortical structures involved in cognitive functions is the thalamus. The thalamus is a small diencephalic structure located bilaterally near the center of the brain in close proximity to the basal ganglia (Fig. 1A). As illustrated in Fig. 1B, each thalamus consists of multiple nuclei, in total 15 in the automated anatomic labelling atlas 3 (AAL3) (Rolls et al. 2020), although there are discrepancies between specific atlases due to parcellation differences (histological, structural or functional MRI, etc.) and inclusion or exclusion of very small or difficult-to-parcellate nuclei. The thalamic nuclei can be grouped anatomically into four major groups: anterior, medial, lateral and posterior (Kumar et al. 2023). These thalamic nuclei have long been known to act as relay stations between the body and the cerebral cortex, transferring and filtering sensorimotor information, and regulating sleep and wakefulness (Cassel and de Vasconcelos 2015; Torrico and Munakomi 2019). However, this structure has been increasingly associated with higher cognitive functions including language, as well.

Lesions in the thalamus, mostly in the left hemisphere, have long been linked to what is known as thalamic aphasia with symptoms primarily including naming deficits (problems of word finding and retrieval such as semantic paraphasia) with relatively preserved repetition, fluency and grammar (Crosson 2013; De Witte et al. 2011; Nadeau and Crosson 1997), while these language impairments frequently co-occur with symptoms in other domains such as amnestic problems, executive dysfunctions and behavior and/or mood alterations (De Witte et al. 2011). This involvement of the thalamus with language processing is consistent with the literature on functional imaging of the thalamus, a review of which correlated thalamic activation mostly with language production tasks (e.g., word or sentence production) and naming, with stronger activation in the left than the right thalamus (Llano 2013). Consistently, an extensive review of neuroscience research also associated the thalamus with lexical, semantic, and prosodic processing; category-specific naming; speech production; linguistic integration, among other cognitive functions (Janacsek et al. 2022). Still other language functions attributed to the thalamus include a role in syntactic structure building (syntactic unification) and syntactic and semantic analysis (detection of syntactic and semantic violations) (David et al. 2011; Friederici et al. 2009; Liu et al. 2023; Wahl et al. 2008), and serving as a central monitor for language-related cortical activations during comprehension and production (Klostermann et al. 2013).

### Structural and functional connectivity between thalamus and cortex

Given the aforementioned associations between the thalamus and cognitive functions including language, several studies sought to reveal structural and functional connections between the thalamus and cortical sites that have been more traditionally linked to those cognitive functions. Structural connections have been typically investigated in vivo using diffusion-weighted tractography (DWT), which involves a special use of MRI to identify white matter fibers by measuring diffusion of water molecules (Alexander et al. 2007; van den Heuvel and Hulshoff Pol 2010), while functional connectivity has been frequently studied using resting-state fMRI (rsfMRI), which estimates spontaneous low-frequency fluctuations in the BOLD signal (Lee et al. 2013; van den Heuvel and Hulshoff Pol 2010). It is generally assumed that functional connections strongly correlate with structural white matter connections (Greicius et al. 2009; Huang and Ding 2016; van den Heuvel and Hulshoff Pol 2010). This section briefly reviews previous DWT and rsfMRI studies that examined structural and functional connectivity patterns of the thalamus.

DWT studies revealed structural connections between the anterior, medial, posterior and lateral nuclei groups of the thalamus and various subcortical and cortical sites including the prefrontal, parietal and temporal cortices (Bohsali et al. 2015; Cunningham et al. 2017; Ford et al. 2013; Grodd et al. 2020; Jeon et al. 2014; Kumar et al. 2023). Of these studies, some have particularly associated the anterior nuclei of the thalamus with the limbic and memory systems through their connections with other diencephalic structures (hypothalamus), medial temporal lobe structures (amygdala, hippocampus) and prefrontal cortex (Grodd et al. 2020), while others have linked the thalamus with the default mode network through its connections with the hippocampus, medial prefrontal cortex, superior frontal gyrus and the precuneus (Cunningham et al. 2017). In studies more specifically examining tracts connecting language-related regions, Broca’s area (pars opercularis, pars triangularis) as well as other prefrontal regions were found to structurally connect especially to the ventral anterior nucleus of the thalamus (Barbas et al. 2013; Bohsali et al. 2015; Ford et al. 2013; Jeon et al. 2014). Other thalamic nuclei that exhibited structural connections with Broca’s area and other prefrontal regions include the pulvinar in the posterior nuclei group (Bohsali et al. 2015) and the medial dorsal nucleus (Barbas et al. 2013; Jeon et al. 2014). It has been suggested that the connections between Broca’s area and the thalamus (ventral anterior nucleus, pulvinar) constitute a cortico-thalamo-cortical loop that plays a role in lexical-semantic processing (Bohsali et al. 2015; Crosson 2021). In particular, the thalamus is argued to mediate transfer of semantic (pars triangularis/orbitalis) and phonological (pars opercularis) information between these anterior/posterior subdivisions of Broca’s area during lexical-semantic processing, given also previous research associating thalamic aphasia with word finding problems and semantic paraphasias (Bohsali et al. 2015; Crosson 2021). In some of those DWT studies, prefrontal regions including Broca’s area which are structurally connected to the thalamus were found to connect also to the basal ganglia structures including the putamen (Ford et al. 2013) and the caudate body/head (Jeon et al. 2014). This cortico-striatal-thalamo-cortical loop was also associated with lexical-semantic processing, with the cortico-striatal part of the network serving to enhance the activation of contextually most suitable semantic/phonemic representations while inhibiting competitors (Ford et al. 2013).

Investigations of the thalamus using rsfMRI identified functional connectivity patterns across the cortex largely overlapping with its structural connections (Cunningham et al. 2017; Hale et al. 2015; O’Muircheartaigh et al. 2015), but also with some differences mainly due to certain medial group nuclei projecting to the cortex indirectly via subcortical connections while their functional connectivity involves all cortical lobes (Kumar et al. 2017). Furthermore, clinical imaging studies also revealed altered structural and functional thalamocortical connections in various neurological conditions. For instance, decreased prefrontal-thalamic structural and functional connectivity was associated with cognitive impairments in Schizophrenia (Giraldo-Chica et al. 2018; Giraldo-Chica and Woodward 2017). Likewise, altered resting-state functional connectivity between the thalamus and cortex has been observed in mild cognitive impairment and Alzheimer’s disease (Wang et al. 2012; Zhou et al. 2013). These alterations mainly include reduced connections of the thalamus with precentral and parietal cortices, as well as increased connections in a host of cortical sites, interpreted as thalamocortical network disconnection and compensatory mechanisms, respectively (Wang et al. 2012; Zhou et al. 2013). Other conditions with altered structural and/or functional thalamo-cortical connections include autism (Tomasi and Volkow 2019), developmental dyslexia (Müller-Axt et al. 2017; Tschentscher et al. 2019), and age-related cognitive decline (Fama and Sullivan 2015). Finally, altered resting-state functional and effective connectivity as well as task-based effective connectivity was observed in the basal ganglia (putamen, caudate nucleus) - thalamo - cortical [(pre) supplementary motor cortex, primary motor cortex, inferior frontal gyrus, middle and superior temporal gyri] circuit in developmental stuttering, pointing to dysfunctional circuits that support speech planning and timing cues necessary to initiate and execute motor sequences (Lu et al. 2010; Qiao et al. 2017). These associations highlight the various functions that the thalamus assumes in various cognitive domains.

As briefly reviewed here, previous explorations of structural and functional connectivity of the thalamus demonstrated cortico-thalamic as well as cortico-striatal-thalamic networks which may underlie various functions likely including linguistic ones. However, these techniques are not without limitations, which may limit the conclusions drawn. For example, the limited resolution of DWT based on the currently available models make it difficult to link white matter fibers to specific grey matter sites (Ford et al. 2013; Kumar et al. 2017; Xiang et al. 2010). Indeed, a comparison of DWT and rsfMRI of the thalamus suggests that structural connections identified with DWT do not align well with rsfMRI connections and that due to its methodological limitations (spatial resolution, fiber crossings, etc.), DWT is of limited use in reflecting functional variability in corticothalamic connections (Kumar et al. 2017). Moreover, the extent to which functional and structural connections identified in these studies are language-relevant is not clear as DWT and rsfMRI detect task-independent connectivity patterns which may not apply, to the same degree, to different cognitive domains or functions. In other words, language tasks may engage certain thalamocortical connections more than others. Finally, although effective connectivity can be used in a task-dependent manner to reveal causal associations among brain regions, it is usually carried out with small sample sizes and for specific tasks, limiting generalizability of findings across populations and tasks within a functional domain.

A recent development in neuroimaging research which can potentially overcome the aforementioned limitations of structural, resting-state and effective functional connectivity techniques is meta-analytic connectivity modeling (MACM). Using a database of functional neuroimaging experiments (BrainMap), MACM can compute functional convergence of studies that report activation in a predetermined region of interest (ROI) in a task-independent manner; i.e., across all behavioral domains available in the database, (Erickson et al. 2017; Robinson et al. 2010) or task-dependent manner; i.e., for a specific behavioral domain such as language (Ardila et al. 2016; Bernal et al. 2015; Viñas-Guasch and Wu 2017). Because a broad database of experiments with various tasks and experimental designs are pooled, MACM can yield highly generalizable findings (Samartsidis et al. 2020).

MACM has been used to study language-related coactivation patterns of cortical and subcortical sites potentially relevant for language, including the left IFG (Bernal et al. 2015; Bulut 2022), right IFG (Bulut 2022), Wernicke’s area (Ardila et al. 2016) and putamen (Viñas-Guasch and Wu 2017). Recent MACM investigations of IFG revealed a language network spanning largely left-lateralized frontal, temporal and parietal regions as well as several subcortical structures (Bernal et al. 2015; Bulut 2022). These subcortical structures were the thalamus, putamen and the cerebellum, which coactivated with the left pars opercularis (BA44) (Bernal et al. 2015; Bulut 2022). When the coactivation patterns of the IFG subdivisions (pars opercularis, pars triangularis, pars orbitalis) were compared in the left hemisphere, only the left pars opercularis was found to significantly coactivate with the left basal ganglia (putamen), left thalamus (medial dorsal nucleus) and the right cerebellum (culmen) (Bulut 2022). Likewise, in the right IFG, significant coactivation with the left thalamus (anterior nucleus) was found only for the right pars opercularis (Bulut 2022). Despite these studies examining language-related coactivation patterns of mainly cortical regions, no MACM of the thalamus was conducted before and no large-scale language-related functional connectivity of the thalamus was carried out. Indeed, the thalamus, which has been shown to influence whole-brain activity, has not been sufficiently explored in the human neuroimaging literature, making it hard to attribute specific functions to it (Shine et al. 2023).

Against this background, the present study utilizes MACM to investigate language-related coactivation patterns of the left and right thalami using a large-scale dataset. To accomplish this, we searched the BrainMap functional database to identify neuroimaging experiments reporting language-related activations in the left and right thalami, separately. The identified activation foci were then subjected to activation likelihood estimation (ALE) analyses to determine which areas significantly coactivated with the left and right thalami. In addition to MACM, a functional decoding analysis was performed for the same areas using the same database in order to characterize linguistic functions of the areas, which could then help interpret the functional profiles of the networks identified. Identification of language-related coactivation patterns of the thalamus can provide insights into the cortico-subcortical organization of language and inform neurocognitive models of language processing, which are largely cortico-centric, thereby helping alleviate cortical bias in cognition in general and language in particular. In addition, delineating the thalamocortical network of language processing in health may provide a baseline against which to compare the language network in thalamic aphasia.

## Materials and methods

### Database search

The database searches aimed to identify language-related functional imaging (fMRI or PET) experiments which report whole-brain activations within the left or right thalamus. The searches were carried out within the BrainMap functional database on January 4, 2023 (and repeated on April 18, 2023 with the same number of hits) using Sleuth Version 3.0.4 (Fox et al. 2005; Fox and Lancaster 2002; Laird et al. 2005). At the time when the searches were repeated, the functional database comprised 4173 papers, 21,336 experiments, and 106,520 subjects. The following search criteria were used: “experimental context: normal mapping”, “experimental activation: activations only”, “behavioral domain: cognition-language”. In addition to these experimental criteria, the left and right thalami were separately included among the search terms as regions of interest (ROIs). These ROIs were specified using a combination of Talairach Daemon (TD) labels (Lancaster et al. 1997, 2000) embedded in Sleuth. That is, the left thalamus was specified by the combination of “TD Label - Hemisphere - Left Cerebrum” AND “TD Label - Gyrus - Thalamus”, and the right thalamus was specified by the combination of “TD Label - Hemisphere - Right Cerebrum” AND “TD Label - Gyrus - Thalamus”. Thus, two searches were performed, one for the left thalamus and another one for the right thalamus. The search term “normal mapping” ensured inclusion of only the experiments conducted with healthy subjects.

As the purpose of the searches was to identify language-related coactivations of the ROIs, the searches were restricted to the “cognition-language” behavioral domain only, which comprises all linguistic levels (phonology, orthography, semantics, syntax, speech) available on BrainMap. However, we did not include “action-execution-speech” among the search terms to exclude purely action-related processes of articulation given the involvement of the thalamus in motor planning, execution, and action selection (Fisher and Reynolds 2014; Kumar et al. 2023). Note that the term “action-execution-speech” was not included as an exclusionary criterion, either, meaning that experiments which involved both action-execution-speech and a language subdomain were also identified by the searches. Also note that an experiment may relate to more than one behavioral domain/subdomain (e.g., both cognition-language and perception-audition).

The database searches yielded 129 papers reporting 186 experiments from a total of 1961 participants for the left thalamus, and 88 papers reporting 118 experiments from a total of 1317 participants for the right thalamus. The distribution of the identified experiments across BrainMap language subcategories are summarized in Table 1 (for details on the BrainMap taxonomy, please refer to Fox et al. 2005; Lancaster et al. 2012). Note that for the left and right thalami, there were 4 and 5 experiments, respectively, which were categorized as language but were not assigned to a specific language subcategory. For both ROIs, the language subcategory with the highest number of hits was semantics followed by speech. It should be cautioned, however, that the sheer number of identified experiments per language subcategory or paradigm class (explained below) does not indicate functional specialization within the ROIs for that subcategory or paradigm, as these numbers may have been driven simply by larger representation in the database (e.g., more semantic hits for the ROIs may be due to higher number of experiments in the database that pertain to semantics compared to other subcategories). Therefore, the total number of experiments in the database as well as percentages of ROI hits for each subcategory or paradigm are also provided in the tables below. Also, inferential statistics based on observed and expected fractions are carried out below (Functional decoding) to examine functional specialization for language subcategories. In terms of percentages of hits per subcategory, speech had the highest representation for both ROIs among the subcategories.

**Table 1:** Distribution of the identified experiments across BrainMap language subcategories for each ROI and for the entire database

Domain	Category (Subcategory)	Database total	L thalamus hits		R thalamus hits	
			#	%	#	%
Cognition	Language (Orthography)	355	18	5.1	14	3.9
Cognition	Language (Phonology)	462	21	4.5	12	2.6
Cognition	Language (Semantics)	1519	96	6.3	56	3.7
Cognition	Language (Speech)	1229	92	7.5	53	4.3
Cognition	Language (Syntax)	251	13	5.2	6	2.4

Note: In this and the following table, the # columns indicate the raw number of experiments identified for the relevant ROI for each category, while the % columns indicate the percentage of all the experiments in the database for each category which were identified for the relevant ROI.

The distribution of the identified experiments across BrainMap paradigm classes related to language are summarized in Table 2 for each ROI. In parallel with the language subcategory distributions, the paradigm with the highest number of hits for both ROIs was semantic monitor/discrimination, which is defined on the BrainMap website as “Discriminate between the meanings of individual lexical items or to indicate if target word is semantically related to the probe word.” (http://www.brainmap.org/taxonomy/paradigms/). The paradigm with the second highest number of hits for both ROIs was overt reading. In terms of percentages of hits per paradigm, if we put aside the paradigms with minimal contribution (1–3 hits) to the samples, figurative language had the highest percentage of hits followed by naming (covert) and word generation (covert) for the left thalamus, and, for the right thalamus, the highest was reading (overt) followed by semantic monitor/discrimination and reading (covert).

**Table 2:** Distribution of the identified experiments across BrainMap paradigm classes related to language for each ROI and for the entire database

Paradigm	Database total	L thalamus hits		R thalamus hits	
		#	%	#	%
Affective words	284	3	1.1	1	0.4
Figurative language	39	6	15.4	1	2.6
Imagined objects/scenes	268	1	0.4	1	0.4
Lexical decision	106	3	2.8	3	2.8
Naming (covert)	109	7	6.4	2	1.8
Naming (overt)	334	13	3.9	8	2.4
Orthographic discrimination	303	8	2.6	8	2.6
Passive listening	598	4	0.7	3	0.5
Phonological discrimination	426	14	3.3	10	2.3
Reading (covert)	374	13	3.5	11	2.9
Reading (overt)	347	20	5.8	21	6.1
Recitation/repetition (covert)	60	2	3.3	2	3.3
Recitation/repetition (overt)	139	8	5.8	2	1.4
Semantic monitor/discrimination	974	53	5.4	31	3.2
Syntactic discrimination	87	5	5.7	3	3.4
Visual object identification	673	2	0.3	1	0.1
Word generation (covert)	304	19	6.3	8	2.6
Word generation (overt)	346	19	5.5	8	2.3
Word stem completion (covert)	10	1	10.0	1	10.0
Word stem completion (overt)	35	1	2.9	1	2.9
Writing	27	2	7.4	1	3.7

Some of the tasks used by the identified experiments involved a button press or a hand movement including a lexical decision task, orthographic, semantic, and syntactic discrimination tasks, and writing. In order to minimize motoric confounds due to hand movement, which typically involves the right hand, we identified experiments which did not control for such movement in the baseline task for both the left and right thalami and performed robustness analyses without these confounded experiments in addition to the main analyses. In other words, we excluded experiments where the main task involves hand movement such as a button press and the control task involves a low-level baseline without a corresponding hand movement such as fixation or rest. We thus identified 23 experiments (16 papers) for the left thalamus, and 20 experiments (11 papers) for the right thalamus as confounding hand movement. Hence, the robustness analysis of the left thalamus included 163 experiments (113 papers), and that of the right thalamus included 98 experiments (77 papers) which had appropriate baselines to control for hand movement.

The foci identified in each search were grouped using the most conservative approach (Turkeltaub et al. 2012); i.e., foci reported in multiple experiments from a single study were combined and entered into the meta-analyses as a single experiment to avoid overinfluence of a single experiment on the results. Coordinates reported in Talairach space were automatically converted into MNI space using the icbm2tal transform (Laird et al. 2010; Lancaster et al. 2007).

### MACM analyses

The activation coordinates identified for each ROI were entered in the ALE analyses, which were run on GingerALE 3.0.2 (Eickhoff et al. 2009, 2012) to compute convergence of coactivations for each ROI. These analyses were run separately with all the experiments identified in the database (main analyses) and with the experiments that had appropriate baselines to control for hand movement (robustness analyses) as explained in the preceding section. The ALE analyses were performed in accordance with the standard procedures reported in previous research (Cieslik et al. 2015; Müller et al. 2017; Wojtasik et al. 2020). Specifically, using a full-width half-​maximum based on the sample size in each experiment, 3D Gaussian probability distributions centered at each foci group were generated (Eickhoff et al. 2009). Then, the union of modeled activation maps was obtained to calculate voxel-wise ALE scores. Next, the union of these activation probabilities were compared against the null hypothesis of random spatial association between the experiments. Finally, the *p*-value distributions derived from these probabilities were thresholded at a voxel-level uncorrected cluster-forming threshold of *p* < 0.001 and a cluster-level corrected threshold of *p* < 0.05 (family-wise error-corrected for multiple comparisons), with 10,000 thresholding permutations.

Following the coactivation analyses separately for each ROI, the conjunction/intersection of coactivation (L thalamus ∩ R thalamus) was visualized using the “Overlay Logicals” utility of the Mango software (Lancaster et al. 2010). The Talairach Daemon embedded in GingerALE was used to generate anatomical labels as the nearest gray matter within 5 mm for the activation peaks (Lancaster et al. 1997, 2000). The Mango software (Lancaster et al. 2010) was used to visualize the ALE results, which were overlaid on the MNI template Colin27_T1_seg_MNI.nii (Kochunov et al. 2002) downloaded from the GingerALE website. The Sleuth files (workspace files including metadata of the experiments identified in each search, and text files containing the foci obtained from the identified experiments and entered in the meta-analyses) as well as the GingerALE output files along with coactivation peaks and cluster analyses for each ROI are available at the online data repository.

### Functional decoding

**Fig. 2:**
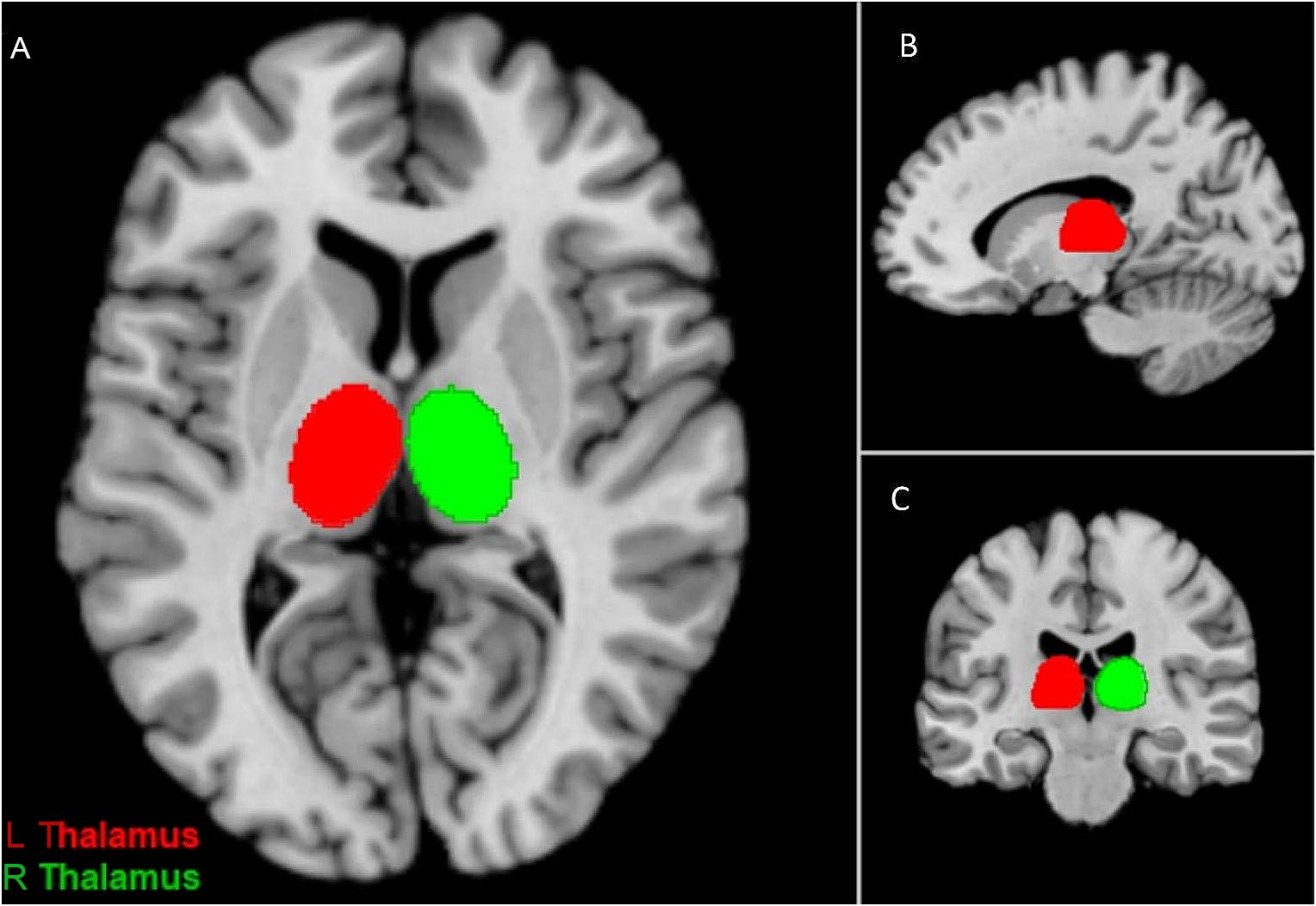
ROIs defined for the functional decoding analysis and shown on axial **(A)**, sagittal **(B)** and coronal **(C)** planes. The ROIs can be downloaded from the online data repository. Also see the online data repository for a video of the animated axial and coronal slices illustrating the ROIs.

Functional decoding of the ROIs was carried out using the Mango Behavioral Analysis Plugin (Lancaster et al. 2012), which was used in previous research to examine functional specialization in various brain regions (Erickson et al. 2017; Sundermann and Pfleiderer 2012). The plugin utilizes the metadata of articles in the BrainMap database to calculate the observed fraction of activation coordinates for a given behavioral subdomain (e.g., cognition.attention, or cognition.language.phonology) that fall within a prespecified ROI and compares it to the fraction that would be expected if the distribution was random. If the difference between the observed and the expected fraction is high, then the ROI is associated with this behavior. In the original description of the Mango Plugin, the statistical threshold for significance is determined as a Z-score ≥ 3.0, which corresponds to a one-tailed (testing only positive association) *p*-value of 0.05, Bonferroni corrected for 51 behavioral subdomains in BrainMap (Lancaster et al. 2012). However, since the purpose of the functional decoding procedure carried out in the present study was to characterize linguistic functions of each ROI, rather than testing their domain-specificity (e.g., language vs. working memory), the analysis was restricted to the five language subdomains available on BrainMap (orthography, phonology, semantics, speech and syntax). Hence, in the present study, the statistical threshold was set as Z > 2.33 (corresponding to a one-tailed *p*-value of 0.05, Bonferroni corrected for the five language subdomains examined). The functional decoding analysis was performed on the same date as the database search, again using the left and right thalami as ROIs. For functional decoding, the ROIs were defined based on the Automated Anatomical Labeling (AAL) atlas (Tzourio-Mazoyer et al. 2002) and using MRIcron (https://www.nitrc.org/projects/mricron) (Rorden and Brett 2000), as illustrated in Fig. 2 (the ROIs can be downloaded from the online data repository). The sizes of the left and right thalamic ROIs were 7699mm^3^ and 7762mm^3^, respectively. The ROIs were visually inspected using the Talairach Daemon in Mango (Lancaster et al. 1997, 2000) and using different brain templates in MRIcron, including AAL3.1 (Rolls et al. 2020), AICHA (Joliot et al. 2015), and the Harvard-Oxford Subcortical Atlas (Desikan et al. 2006; Frazier et al. 2005) to ensure that the intended brain regions were captured and that the parcellation was consistent across other subcortical atlases, which was the case.

## Results

### MACM main results

Chi-square tests were carried out to examine whether the number of papers and experiments identified for the left and right thalami differed significantly. It was found that significantly more papers (*X*^2^ > 7.747, *p* = 0.005) and experiments (*X*^2^ > 15.211, *p* < 0.001) were identified for the left than right thalamus. This finding shows that more studies in the BrainMap database reported activations in the left than right thalamus for language tasks.

**Fig. 3:**
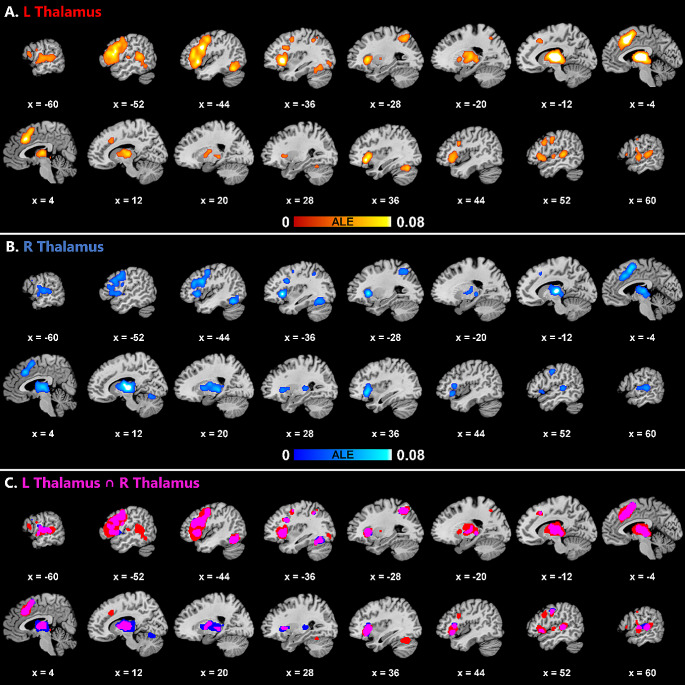
Coactivation results for the left thalamus **(A)** and the right thalamus **(B)**, and the intersection of the coactivations for the left and right thalami (left thalamus in red, right thalamus in blue, intersection in magenta) **(C)**

The coactivation and intersection results for each ROI are visualized in Fig. 3 and the coactivation results are summarized in Table 3. As can be seen from comparison of the coactivation maps (Fig. 3A and B), the left thalamus showed more extensive coactivation (total cluster size = 122,232mm^3^) than the right thalamus (total cluster size = 84,456mm^3^). Despite this difference, the ROIs coactivated with a generally overlapping network of regions (Fig. 3C). Both ROIs significantly coactivated with mainly left-lateralized cortical clusters spanning the frontal, temporal and, to a limited extent, parietal lobes, as well as largely bilateral subcortical and right cerebellar clusters.

For both ROIs, the left frontal clusters included the insula, inferior frontal gyrus peaking at BA44 and 9, but also spanning parts of BA45 and 47, middle frontal gyrus, precentral gyrus and medial frontal regions. The right frontal coactivations were limited compared to the left-hemispheric ones, however, involving the right insula and precentral gyrus for both ROIs, and the right inferior frontal gyrus (peaking at BA9, but also spanning parts of BA44, 45 and 47) for the left thalamus.

As for the temporal coactivations, both ROIs coactivated with parts of the primary auditory cortex including BA41 and 42. The ROIs also coactivated with the association cortex of the middle and superior temporal lobe bilaterally, including BA22, and with the left fusiform gyrus. As can be seen in the conjunction analysis (Fig. 3C), particularly the left thalamus coactivated with the posterior superior/middle temporal cortex bilaterally with greater involvement of the left hemispheric regions overlapping with Wernicke’s area. Within the parietal lobe, both ROIs coactivated mainly with the left superior parietal lobule including the precuneus, while this cluster also spanned parts of the left inferior parietal lobule for the left thalamic ROI.

Subcortically, both ROIs coactivated with themselves as well as the homotopic thalamus in the contralateral hemisphere, including the medial dorsal nucleus and the ventral dorsal nucleus. Other subcortical peaks included the claustrum and the right basal ganglia (the left thalamus coactivating with the right lateral globus pallidus, and the right thalamus coactivating with the right putamen). In addition, the subcortical clusters spanned parts of the bilateral striatum (putamen and caudate body) and bilateral pallidum (lateral globus pallidus) of the basal ganglia for both the left and right thalami. Finally, both ROIs exhibited significant coactivations with the right cerebellum. The left thalamus coactivated mostly with the anterior lobe (culmen) (56.5% of the cerebellar cluster), but also with the posterior lobe (declive, tuber, uvula, pyramis) (43.5%) of the right cerebellum, while the right thalamus coactivated mostly with the posterior lobe (declive) (97.6%), but, to a limited extent, also with the anterior lobe (culmen) (2.4%) of the right cerebellum.

**Table 3:** Coactivation results for the left and right thalami

Cluster	Anatomical Label (Nearest Gray Matter within 5 mm)	BA	MNI Coordinates			ALE	Z	Cluster Size (mm^3^)
			x	y	z			
L thalamus								
1	L Insula	13	-34	22	0	0.113	10.04	39,160
	L IFG	9	-44	10	28	0.092	8.46	
	L IFG	9	-50	10	20	0.086	8.02	
	L IFG	44	-52	16	12	0.081	7.60	
	L MFG	46	-48	26	16	0.080	7.55	
	L Precentral gyrus	6	-46	-2	44	0.073	6.96	
	L Precentral gyrus	6	-50	-10	34	0.051	5.01	
	L Precentral gyrus	6	-54	-6	24	0.045	4.37	
	L Postcentral gyrus	43	-58	-16	22	0.035	3.33	
2	L Medial dorsal nucleus (Thalamus)		-8	-16	6	0.183	14.83	29,008
	R Medial dorsal nucleus (Thalamus)		10	-18	6	0.073	6.96	
	R Ventral lateral nucleus (Thalamus)		14	-8	10	0.064	6.15	
	R Thalamus		22	-28	0	0.048	4.69	
	R Lateral globus pallidus		18	0	2	0.040	3.86	
3	L Fusiform gyrus	37	-42	-60	-18	0.077	7.29	14,728
	L MTG	22	-54	-42	6	0.065	6.25	
	L STG	41	-58	-16	6	0.053	5.14	
	L IOG	19	-38	-80	-6	0.043	4.17	
	L MTG	20	-54	-46	-12	0.037	3.52	
4	L FGmed	32	-2	14	46	0.116	10.25	14,576
5	R Insula	13	36	26	-8	0.087	8.09	9488
	R Claustrum		36	20	-2	0.077	7.27	
	R IFG		50	18	-4	0.055	5.33	
6	L SPL	7	-26	-62	48	0.062	5.96	3896
7	R STG	22	54	-32	4	0.068	6.48	3624
8	R Culmen (Cerebellum, anterior lobe)		36	-66	-26	0.063	6.05	2464
	R Culmen (Cerebellum, anterior lobe)		34	-58	-26	0.050	4.85	
	R Culmen (Cerebellum, anterior lobe)		26	-54	-22	0.039	3.74	
9	R STG	22	58	-10	-2	0.053	5.18	2000
	R Postcentral gyrus	43	62	-6	12	0.039	3.74	
10	R IFG	9	48	10	28	0.062	6.01	1936
11	R Precentral gyrus	4	54	-8	38	0.050	4.87	1352
R thalamus								
1	L Thalamus		-12	-18	6	0.105	10.32	35,008
	R Medial dorsal nucleus (Thalamus)		12	-18	4	0.105	10.31	
	R Claustrum		34	22	-4	0.073	7.69	
	R Thalamus		22	-28	0	0.062	6.70	
	R Putamen		22	4	2	0.047	5.24	
	L Thalamus		-6	-26	-4	0.045	5.08	
	R Insula	13	40	20	8	0.044	4.93	
	L Thalamus		-20	-30	-2	0.040	4.54	
	R Insula	13	50	14	-4	0.035	3.96	
	R Parahippocampal gyrus	30	18	-38	8	0.029	3.32	
2	L Claustrum		-32	20	-2	0.104	10.27	25,736
	L IFG	9	-42	10	26	0.069	7.36	
	L IFG	47	-46	20	-2	0.059	6.38	
	L MFG	46	-44	26	22	0.053	5.83	
	L Precentral gyrus	4	-46	-4	46	0.051	5.66	
	L Precentral gyrus	6	-50	2	36	0.050	5.60	
	L IFG	9	-52	12	28	0.048	5.39	
	L STG	41	-60	-20	6	0.045	5.08	
	L Insula	13	-50	10	-2	0.044	4.94	
	L MTG		-58	-32	2	0.039	4.38	
	L STG	22	-56	-8	-2	0.033	3.77	
	L IFG	44	-54	18	12	0.033	3.76	
	L Precentral gyrus	6	-56	-4	24	0.032	3.65	
	L TTG	42	-60	-10	14	0.032	3.60	
3	L FGmed	6	-2	10	50	0.079	8.20	10,376
	L Cingulate gyrus	32	0	20	38	0.070	7.44	
4	L Fusiform gyrus	37	-42	-60	-18	0.064	6.91	4800
5	R STG	22	58	-28	4	0.052	5.77	3608
	R STG	22	60	-10	2	0.033	3.72	
6	L SPL	7	-26	-62	48	0.046	5.18	2792
	L SPL	7	-30	-52	48	0.043	4.81	
7	R Precentral gyrus	4	54	-8	38	0.043	4.85	1120
	R Precentral gyrus	6	48	2	42	0.031	3.50	
8	R Declive (Cerebellum, posterior lobe)		12	-66	-14	0.039	4.41	1016

Note: MNI Coordinates correspond to cluster peaks, and anatomical labels indicate gray matter nearest to the cluster peaks. Please refer to the online data repository for cluster analyses with full reports of structures included in each cluster. L: Left, R: Right, FGmed: Medial frontal gyrus, IFG: Inferior frontal gyrus, IOG: Inferior Occipital Gyrus, MFG: Middle frontal gyrus, MTG: Middle temporal gyrus, SPL: Superior parietal lobule, STG: Superior temporal gyrus, TTG: Transverse temporal gyrus

### MACM robustness results

This section summarizes the results of the analyses conducted with the experiments that had appropriate baselines to control for hand movement. The robustness coactivation and intersection results for each ROI are visualized in Figure S1 and the coactivation results are summarized in Table S1 in the Supplementary Information. The pattern of coactivation results in the robustness analyses was largely similar to the pattern of results in the main analyses. In particular, both thalami coactivated mainly with bilateral frontotemporal regions and bilateral subcortical regions (i.e., basal ganglia and thalamus). The primary difference between the main results and the robustness results involved the coactivations of the right thalamus, which were reduced in the robustness analyses (a total cluster size reduction of 45.0% compared to the main results) particularly in the left hemisphere and which did not extend to the cerebellum contrary to the main results. This suggests that at least part of the left cortical and subcortical, as well as all right cerebellar, coactivations of the right thalamus in the main results may have been driven by hand movement during task performance. The coactivations of the left thalamus, however, were not reduced to such an extent in the robustness analyses (a total cluster size reduction of 8.9% compared to the main results) and, despite being bilateral, still exhibited left dominance in the cortex and subcortex, and spanned the right cerebellum, as in the main results. Taken together, the robustness results largely support the main results that the language-relevant coactivation network of the thalamus involves bilateral frontotemporal and bilateral subcortical regions for both the left and right thalami, and also the right cerebellum for the left thalamus, even after controlling for potential confounds due to hand movement.

### Functional decoding results

**Fig. 4:**
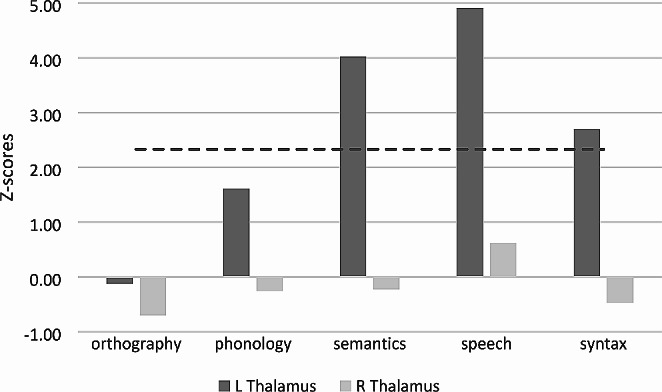
Functional decoding results of the ROIs for the five language subdomains in BrainMap (the dashed line represents the statistical threshold of Z > 2.33, which corresponds to a one-tailed *p*-value of 0.05, Bonferroni corrected for the five language subdomains examined)

The functional decoding results for the left and right thalami are illustrated in Fig. 4. The results showed that the left thalamus was significantly associated with speech (Z = 4.91), semantics (Z = 4.02) and syntax (Z = 2.70). The right thalamus, on the other hand, was not significantly associated with any language subdomains (Z < 0.62).

## Discussion

The meta-analytic connectivity modeling of the left and right thalami identified bilateral frontotemporal and bilateral subcortical coactivations for both regions of interest, and right cerebellar coactivations for the left thalamus, during language tasks. The distribution of experiments included in the analyses across language subcategories and paradigm classes and the functional decoding analysis showed that thalamic activation was associated mostly with language production/speech, semantics and syntax. Taken together with previous empirical studies and theoretical frameworks, the findings suggest that cortico-subcortical-cerebellar-cortical loops modulate and fine-tune information transfer within the bilateral frontotemporal cortices during language processing.

Cortically, the language-related coactivation patterns of both the left and right thalami spanned frontotemporal regions, distributed bilaterally but stronger in the left hemisphere for the left thalamus, which were previously associated with language functions. Although both ROIs showed left parietal coactivations mainly within the superior parietal lobule, the parietal coactivations of only the left thalamic ROI survived the robustness analyses. These findings are consistent with previous DWT and rsfMRI investigations of the thalamus which revealed structural and functional connectivity between the thalamus and mainly frontotemporal cortical sites (Bohsali et al. 2015; Cunningham et al. 2017; Ford et al. 2013; Grodd et al. 2020; Hale et al. 2015; Jeon et al. 2014; Kumar et al. 2023; O’Muircheartaigh et al. 2015). In the present study, the frontal coactivations spanned bilaterally the precentral gyrus, insula and the inferior frontal gyrus for both ROIs. The IFG coactivations spanned subdivisions of the Broca’s complex including BA44, 45 and 47 bilaterally but greater in the left hemisphere and for the left thalamus. In parallel with these findings, previous research on structural connectivity of the thalamus with language-related regions linked the thalamus (ventral anterior nucleus, medial dorsal nucleus, pulvinar) with Broca’s area (BA44, 45) as well as other prefrontal regions (Barbas et al. 2013; Bohsali et al. 2015; Ford et al. 2013; Jeon et al. 2014). These connections constitute a cortico-thalamo-cortical loop, which is argued to play a role in lexical-semantic processing (Bohsali et al. 2015; Crosson 2021). In this framework, the thalamus mediates transfer of semantic and phonological information between BA45 and 44 during lexical-semantic processing (Bohsali et al. 2015; Crosson 2021). The present findings suggest that this loop may extend to BA47 as well, given also previous research implicating this area with semantic processes (Binder et al. 2009; Hagoort and Indefrey 2014; Rodd et al. 2015) and with interface functions between semantic and limbic networks (Belyk et al. 2017).

In the temporal lobe, the coactivation networks of both ROIs included the primary auditory cortex as well as the association cortex of the middle and superior temporal lobe including BA22 bilaterally. In particular, coactivations were seen in the left posterior superior and middle temporal gyri, overlapping with regions traditionally considered as Wernicke’s area, especially for the left thalamus. These posterior superior/middle temporal cortices were previously associated with various linguistic functions, primarily including phonological processing (particularly the superior temporal gyrus) (Turkeltaub and Branch Coslett 2010; Vigneau et al. 2006), syntactic processing (Friederici 2012; Hagoort and Indefrey 2014; Heard and Lee 2020; Rodd et al. 2015; Vigneau et al. 2006; Walenski et al. 2019) and semantic processing (Friederici 2012; Hagoort and Indefrey 2014; Rodd et al. 2015; Vigneau et al. 2006). Predominance of the semantic and speech subcategories and semantic and production tasks in the distribution of the pooled experiments (see Tables 1 and 2) and the significant involvement of the left thalamus with speech, semantics and syntax in the functional decoding analysis suggest that the observed coactivation patterns underlie these language functions in particular.

Further, the frontotemporal coactivation patterns of the thalamus found here are compatible with the Memory, Unification and Control (MUC) Model, which associates the left temporal lobe with memory processes and the left IFG with unification processes underlying language (Hagoort 2005, 2013, 2016). According to this view, the memory areas within the left temporal lobe embody the mental lexicon by storing lexical information, while the unification areas within the left IFG are responsible for combinatorial processing of linguistic information. Executive control processes such as attention, selection, turn-taking in conversation and code-switching engaged in language processing are subserved by the control network spanning the dorsolateral prefrontal cortex; i.e., the middle frontal gyrus (BA46, 9, 8, 6), the anterior cingulate cortex and parts of the parietal cortex (Hagoort 2005, 2013, 2016; Xiang et al. 2010). The MUC Model emphasizes dynamic interaction between the memory, unification and control processes. Importantly, gradients within the frontotemporal regions are proposed that map phonological processes dorsally, semantic processes ventrally and syntactic processes in between these ventral and dorsal sites (Hagoort 2013, 2016; Xiang et al. 2010). Specifically, the model associates left BA44/6 and the left posterior superior temporal gyrus/sulcus with phonological processing, left BA45/44 and the left posterior middle temporal gyrus with syntactic processing, and left BA47/45 and the left inferior temporal cortices with semantic processing. As these cortical sites largely overlap with the frontotemporal coactivations of the thalamus observed here, it is possible that indirect cortico-thalamo-cortical loops regulate transfer of information via the direct cortico-cortical circuits within the frontotemporal system.

In particular, cortico-thalamo-cortical, or transthalamic, loops with driver inputs from cortical layer 5 to higher-order thalamus (e.g., the pulvinar and the ventral anterior nucleus) have been suggested to regulate functional connectivity within and across cortical regions and, thus, to be critical for communication amongst cerebral networks underlying various functions including language (Antunes and Malmierca 2021; Crosson 2021; Shepherd and Yamawaki 2021; Sherman 2016; Usrey and Sherman 2019). For instance, in Crosson’s recurrent circuit model, cortico-thalamo-cortical loops support the direct cortico-cortical circuits during word retrieval and other language functions (Crosson 2021). According to the model, during word retrieval, the cortico-thalamo-cortical loop helps maintain stable representations of semantic information at the semantic-lexical interface, which are then compared with emerging lexical alternatives that represent the semantic concept. Thus, the cortico-thalamo-cortical loop provides an error signal for cortico-cortical mechanisms regarding semantic-lexical pairings until a good lexical match has been found for the semantic concept (Crosson 2021). When this error signal is compromised through lesion of the relevant left thalamic nucleus, this results in visual-semantic errors during word retrieval, which is typical of thalamic aphasia (Crosson 2021). Hence, the model proposes a division of labor between direct cortico-cortical and indirect cortico-thalamo-cortical loops, such that the former allows for change necessary to progress to higher-order stages of information processing, while the latter tends to maintain current activity patterns necessary to generate error feedback to the lower-order cortex (Crosson 2021; Kawaguchi 2017). It is further claimed that oscillatory synchrony between the thalamic relays and the cortex in the gamma band is a neural signature for information processing in these two circuits (Crosson 2021). The functional role of these cortico-thalamo-cortical loops during language processing may extend beyond lexical-semantic processes to include unification and control operations at multiple language levels (e.g., syntax, morphology, phonology) which may be shared with other cognitive domains/networks (e.g., theory of mind, attention) through recruitment of bilateral cortical, bilateral subcortical and right cerebellar regions not classically associated with language functions (Hagoort 2016, 2019).

The coactivation patterns of the left and right thalami did not only entail cortical regions, but also subcortical structures including the bilateral basal ganglia and, for the right thalamic ROI, the right cerebellum. In particular, the coactivations of both ROIs spanned parts of the bilateral striatum (putamen and caudate body) and bilateral pallidum (lateral globus pallidus) of the basal ganglia. Previous functional imaging, animal model and clinical studies linked the basal ganglia (usually the putamen and the caudate nucleus) with diverse emotional, motor and cognitive processes (Albin et al. 1989; Chakravarthy et al. 2010; Groenewegen 2003; Lanciego et al. 2012; Packard and Knowlton 2002). Along these lines, the basal ganglia-thalamo-cortical circuitry has been implicated in a range of sensorimotor, cognitive, emotional and motivational brain functions (Alexander and Crutcher 1990; Copland and Angwin 2019; Groenewegen 2003; Murphy et al. 2022). In the domain of language, the left putamen was found to coactivate with areas closely linked to language primarily including frontotemporal regions and these coactivations were associated mainly with semantic processes (Viñas-Guasch and Wu 2017). The left caudate, too, was involved in language processing (Crinion et al. 2006; Tan et al. 2011; Zou et al. 2012), and lesions in the left caudate nucleus correlated with speech and language impairments following stroke, associating this region with control processes involving speech and language (Grönholm et al. 2016). Previous structural and functional connectivity studies, too, showed that prefrontal regions including Broca’s area which were connected to the thalamus were also linked to the basal ganglia structures including the putamen (Bulut 2022; Ford et al. 2013) and the caudate body/head (Jeon et al. 2014). This cortico-striatal-thalamo-cortical loop was also associated with lexical-semantic processing, with the cortico-striatal part of the circuitry increasing the activation of contextually appropriate semantic/phonemic representations while inhibiting competitors (Ford et al. 2013).

The subcortical coactivations of both the left and right thalami identified here included parts of the right cerebellum in the main results, while the right cerebellar coactivations of only the left thalamus survived the robustness analyses. In particular, the left thalamus coactivated mostly with the anterior lobe (culmen) of the right cerebellum. Traditionally associated with coordination of motor control, the cerebellum, especially its right hemisphere, has more recently been linked to mediation of cognitive functions including language (Murdoch 2010). The right cerebellum has been involved in speech and language processing in neuroimaging research (Booth et al. 2007; Wildgruber et al. 2001). In addition, morphological differences in the cerebellum were observed in children with primary language impairments (specific language impairment) and secondary language impairments (comorbid with autism spectrum disorder) compared to their typically developing peers (Hodge et al. 2010). In another study, people with lesions in the left pars opercularis showed greater activation in the right cerebellum during speech production compared to neurologically intact controls and patient controls with lesions somewhere else in the left hemisphere, which was interpreted as recruitment of a compensatory cognitive control mechanism during speech production (Lorca-Puls et al. 2021). In a further fMRI study comparing language production and comprehension, both cortical (bilateral frontal and temporal sites) and subcortical-cerebellar (left thalamus, bilateral caudate, left putamen and bilateral cerebellum) structures were associated with increasing constituent size of the produced or comprehended items (Giglio et al. 2022). Importantly, these subcortical and cerebellar regions showed greater activation for production than comprehension, which was interpreted as reflecting articulatory requirements in production (Giglio et al. 2022). Furthermore, previous resting-state fMRI and meta-analytic connectivity modeling studies showed that Broca’s and Wernicke’s areas were functionally connected to the basal ganglia, the thalamus and the right cerebellum, hence suggesting a language-related function for these subcortical structures (Bulut 2022; Tomasi and Volkow 2012). In particular, investigation of language-related meta-analytic connectivity of the left and right pars opercularis, triangularis and orbitalis using the same database as the present study revealed subcortical (left thalamus, left putamen) and right cerebellar coactivations only for the left pars opercularis (BA44), suggesting that it may be a major hub in the language network with connections to diverse cortical, subcortical and cerebellar structures (Bulut 2022). Relatedly, previous research emphasized the roles of the basal ganglia and the cerebellum that provide output to the thalamic nuclei including the ventral lateral, ventral anterior, intralaminar and mediodorsal nuclei, which are in turn connected to the premotor and prefrontal cortices, and suggested that this cerebellar-basal ganglia-thalamo-cortical system engages in motor and cognitive aspects of language (Barbas et al. 2013). In this framework, the thalamo-cortical circuitry is gated by the inhibitory thalamic reticular nucleus and modulated by dopamine, a specialization in primates (Barbas et al. 2013).

The functional decoding analysis carried out in the present study significantly associated the left, but not the right, thalamus with speech, semantics and syntax, respectively, but not with phonology or orthography. This functional characterization is in accord with the distribution of paradigm classes (Table 2), which shows greater contribution of experiments from semantic (semantic monitoring/discrimination, naming) and production (word generation, reading, naming) tasks to the coactivation analysis. The association of the left thalamus with semantics is consistent with the literature on thalamic aphasia which occurs usually after left thalamic lesion and involves naming deficits (problems of word finding and retrieval such as semantic paraphasia) with relatively preserved repetition (Crosson 2013; De Witte et al. 2011; Nadeau and Crosson 1997). Consistently, the thalamus (particularly ventral anterior nucleus and the pulvinar) and its connections with the prefrontal structures including Broca’s area have been associated with lexical-semantic processing, with the thalamus mediating transfer of semantic (pars triangularis/orbitalis) and phonological (pars opercularis) information between these anterior/posterior subdivisions of Broca’s area during lexical-semantic processing (Bohsali et al. 2015; Crosson 2021). Also, the association of the left thalamus with speech aligns well with studies showing more activation in the thalamus for language production than comprehension tasks (Giglio et al. 2022). Indeed, a review of literature on functional imaging of the thalamus during language tasks correlated thalamic activation mostly with language production tasks (e.g., word or sentence production) and naming, with stronger activation in the left than the right thalamus (Llano 2013). In this review, thalamic activity was found especially for perceptually challenging tasks, suggesting an attentional role to the thalamus (Johnson and Ojemann 2000; Llano 2013). Other speech-related functions attributed to the thalamus, in concert with the cerebellum and the basal ganglia, include the fine-tuning of timing and sequencing in speaking (Hagoort 2019). Finally, the significant association of the left thalamus with syntax is consistent with studies linking this structure to syntactic structure building and syntactic and semantic analysis (detection of syntactic and semantic violations) (David et al. 2011; Friederici et al. 2009; Liu et al. 2023; Murphy et al. 2022; Wahl et al. 2008). In particular, it has been suggested that the thalamus mediates between early syntactic processes contributing to phrase structure building and late integration processes through its connectivity with the anterior frontotemporal and parietotemporal cortices, respectively (Friederici et al. 2009; Wahl et al. 2008).

### Limitations

Several limitations should be addressed. First, the experiments included in the MACM analyses were pooled from all language subcategories and tasks that reported activation in the ROIs, without specifically testing or contrasting involvement with particular language components (e.g., syntax, semantics, phonology, speech) or tasks/modalities (e.g., comprehension, production), which can influence the coactivation patterns. The reason for this is twofold. First, in this first large-scale language-related functional connectivity investigation of the thalamus, our purpose was to delineate the coactivation patterns of the left and right thalami for the language function in general. Second, restricting the analyses on individual language subcategories would have decreased the number of experiments included in each analysis, thereby substantially decreasing the statistical power for most of those subcategories particularly for the right thalamus, but also for the left. This would aggravate the subcortical bias that may already misrepresent the number of experiments linking the thalamus to language functions. Nevertheless, the present findings should be interpreted with caution as there may be differences in the engagement of the connectivity network of the thalamus for different language components or tasks/modalities. When more language-related studies involving the thalamus become available through expansion of the database used here or inclusion/creation of others, future MACM studies can examine coactivation patterns of the thalamus for different language components and/or tasks.

The second limitation concerns inclusion of the left and right thalami as a whole in the analyses, without subdivision into their constituent nuclei. Given that the thalamus consists of many small thalamic nuclei with potentially different functions, pooling all thalamic activations into single analyses may have influenced the results. Indeed, certain thalamic nuclei such as the pulvinar, ventral anterior, ventrolateral and centromedian nuclei have been highlighted as particularly relevant for higher-order functions such as language (Antunes and Malmierca 2021; Crosson 2021; Llano 2016; Shepherd and Yamawaki 2021; Sherman 2016; Usrey and Sherman 2019). Nevertheless, the clinical and functional imaging literature has not consistently implicated a specific thalamic nucleus with language (Llano 2016), with clinical studies reporting thalamic aphasia following lesions to different nuclei (Crosson 2013; De Witte et al. 2011; Nadeau and Crosson 1997), and functional imaging studies with language tasks reporting peaks of activation in almost all thalamic subregions (Llano 2013). Moreover, the relatively small size of thalamic nuclei in view of the limitations of commonly available MRI scanners (e.g., spatial resolution) and analytical techniques (e.g., smoothing windows) coupled with greater susceptibility of subcortical structures to movement artifacts make it difficult to correlate language/cognition with activation in individual thalamic nuclei (Janacsek et al. 2022; Llano 2016). Finally, we chose not to analyze coactivation patterns of individual thalamic nuclei also for the same reasons mentioned in the previous paragraph; namely, our purpose being delineation of the coactivation patterns of the left and right thalami for the language function in general and increasing statistical power by keeping the number of included experiments at maximum.

Finally, given that the thalamus and other subcortical and cerebellar structures that were found to coactivate with the thalamus in the present study have been associated with motor planning, action execution and selection (Brownsett et al. 2014; Fisher and Reynolds 2014; Giglio et al. 2022; Kumar et al. 2023), the likelihood that the observed coactivations may underlie such motor-related activity should be discussed. We have already addressed motoric activation due to hand movement (e.g., button press) by running robustness analyses which included only the experiments that had appropriate baselines to control for hand movement and which revealed a similar pattern of results to the main analyses, particularly for the left thalamus (see the *MACM robustness results* above and the Supplementary Information). However, articulation-related motoric activity may still have contributed to the present results, given that we did not exclude experiments that did not control articulation-related motoric activity (e.g., overt naming versus a low-level baseline such as fixation). Although we did not include “action-execution-speech” among the search terms to exclude purely action-related processes of articulation, this term was not included as an exclusionary criterion, either, meaning that experiments which involved both action-execution-speech and a language subdomain were also identified by the searches. As above, the main reason for this was to increase statistical power by pooling a high number of language-relevant experiments for both the left and right thalami. In particular, exclusion of such experiments; i.e., language-related experiments that did not control articulation-related motoric activity, would have resulted in removal of experiments on language production. These studies are much less represented in the literature than comprehension studies. Moreover, we consider speech articulation to be an integral part of the language production system. That is why we decided to keep these studies in our meta-analysis.

## Conclusion

The present study demonstrated bilateral frontotemporal and bilateral subcortical (basal ganglia) coactivation patterns for both the left and right thalami, and also right cerebellar coactivation patterns for the left thalamus, during language processing. When considered together with previous empirical studies and theoretical frameworks, the meta-analytic connectivity and functional decoding findings suggest that cortico-subcortical-cerebellar-cortical loops modulate and fine-tune information transfer within the bilateral frontotemporal cortices during language processing, especially during production and semantic operations, but also other language (e.g., syntax, morphology, phonology) and cognitive operations (e.g., attention, cognitive control). Overall, the current findings show that the language-relevant network extends beyond the classical left perisylvian cortices to include bilateral cortical, bilateral subcortical (bilateral thalamus, bilateral basal ganglia) and right cerebellar regions. This extended language-relevant network for the thalamus in health can be used as a benchmark to compare the language network in thalamic aphasia.

## Electronic supplementary material

Below is the link to the electronic supplementary material.

Supplementary Material 1

## Data Availability

All data and analysis files associated with the study are available at the online data repository: 10.17605/OSF.IO/JQNFK
