# Supplementary material for: Contributions of the left and right thalami to language: A meta-analytic approach
Source: Brain Struct Funct. 2024 Apr 16;229(9):2149–66. doi: 10.1007/s00429-024-02795-3 (PMC11611992; doi:10.1007/s00429-024-02795-3)
Supplement: Supplementary file 1 — Supplementary Material 1 [file 429_2024_2795_MOESM1_ESM.docx]

**Supplementary Information**

**for “Contributions of the left and right thalami to language: A meta-analytic approach”**

Talat Bulut^1, 2^, Peter Hagoort^1, 3^

^1^ Max Planck Institute for Psycholinguistics, Nijmegen, the Netherlands

^2^ Istanbul Medipol University, Istanbul, Turkey

^3^ Donders Institute for Brain, Cognition and Behaviour, Radboud University Nijmegen, the Netherlands

Corresponding Author:

Talat Bulut: [buluttalat@gmail.com](mailto:buluttalat@gmail.com)

**MACM robustness results**

This section summarizes the results of the meta-analyses conducted with the experiments that had appropriate baselines to control for hand movement. That is, we excluded experiments where the main task involves hand movement such as button press and the control task involves a low-level baseline without a corresponding hand movement such as fixation or rest. Thus, the analyses did not include 23 experiments (16 papers) for the left thalamus, and 20 experiments (11 papers) for the right thalamus which confounded hand movement. Hence, the results of the robustness analyses presented here included 163 experiments (113 papers) for the left thalamus, and 98 experiments (77 papers) for the right thalamus, with all these included experiments having appropriate baselines to control for hand movement.

The robustness coactivation and intersection results for each ROI are visualized in Figure S1 and tabulated in Table S1 below. In the robustness results, the total cluster size was 111,392mm^3^ for the left thalamus, showing a decrease of 8.9% from the main results reported in the paper. The total cluster size for the right thalamus was 46,424mm^3^ in the robustness results, showing a reduction of 45.0% from the main results.


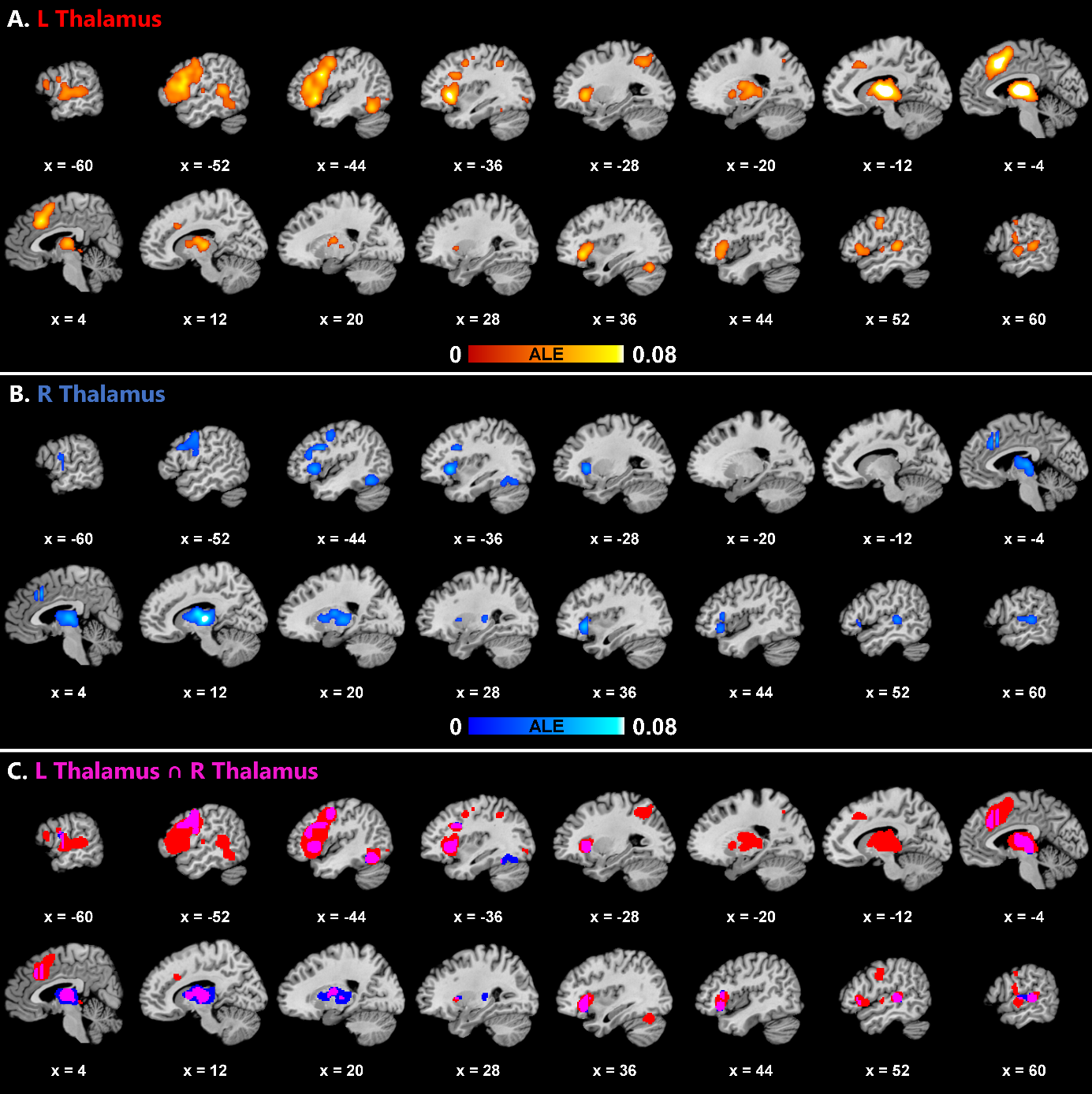


Figure S1. Results of the robustness analyses: Coactivation results for the left thalamus (A) and the right thalamus (B), and the intersection of the coactivations for the left and right thalamus (left thalamus in red, right thalamus in blue, intersection in magenta) (C).

Table S1. Coactivation results for the left and right thalami in the robustness analyses.

| **Cluster** | **Anatomical Label**  (Nearest Gray Matter within 5mm) | **BA** | **MNI Coordinates** | | | **ALE** | **Z** | **Cluster Size** (mm^3^) |
| --- | --- | --- | --- | --- | --- | --- | --- | --- |
|  |  |  | x | y | z |  |  |  |
| L thalamus | | | | | | | | |
| 1 | L Insula | 13 | -34 | 22 | 0 | 0.101 | 9.61 | 51136 |
|  | L IFG | 9 | -42 | 10 | 28 | 0.082 | 8.14 |  |
|  | L Insula | 13 | -42 | 18 | -6 | 0.081 | 8.02 |  |
|  | L Precentral gyrus | 44 | -50 | 14 | 8 | 0.076 | 7.62 |  |
|  | L MFG | 46 | -46 | 28 | 14 | 0.069 | 7.08 |  |
|  | L Fusiform gyrus | 37 | -44 | -60 | -18 | 0.066 | 6.74 |  |
|  | L Precentral gyrus | 6 | -46 | -2 | 42 | 0.064 | 6.56 |  |
|  | L MTG | 22 | -54 | -42 | 6 | 0.060 | 6.27 |  |
|  | L TTG | 42 | -58 | -14 | 6 | 0.049 | 5.23 |  |
|  | L Precentral gyrus | 6 | -52 | -10 | 32 | 0.049 | 5.17 |  |
|  | L Precentral gyrus | 6 | -54 | -6 | 24 | 0.045 | 4.75 |  |
|  | L Fusiform gyrus | 19 | -40 | -80 | -8 | 0.039 | 4.15 |  |
|  | L MOG | 18 | -40 | -84 | -8 | 0.039 | 4.12 |  |
|  | L MTG | 20 | -54 | -46 | -12 | 0.036 | 3.86 |  |
|  | L Precentral gyrus | 4 | -38 | -12 | 50 | 0.034 | 3.62 |  |
| 2 | L Thalamus |  | -8 | -16 | 4 | 0.157 | 1.36 | 26216 |
|  | R Medial dorsal nucleus (Thalamus) |  | 10 | -18 | 6 | 0.064 | 6.58 |  |
|  | R Ventral lateral nucleus (Thalamus) |  | 14 | -8 | 12 | 0.059 | 6.15 |  |
|  | L Lentiform nucleus (Putamen) |  | -16 | 8 | 2 | 0.047 | 4.99 |  |
|  | R Caudate body |  | 12 | 4 | 4 | 0.035 | 3.72 |  |
| 3 | L FGmed | 32 | -2 | 16 | 46 | 0.100 | 9.55 | 14840 |
|  | L Cingulate gyrus | 32 | -4 | 18 | 40 | 0.099 | 9.47 |  |
| 4 | R Insula | 13 | 36 | 26 | -8 | 0.075 | 7.53 | 8392 |
|  | R Insula | 13 | 38 | 20 | 0 | 0.059 | 6.16 |  |
|  | R Precentral gyrus | 44 | 48 | 22 | 2 | 0.048 | 5.04 |  |
|  | R IFG | 45 | 50 | 20 | -2 | 0.047 | 5.04 |  |
|  | R Insula | 13 | 50 | 10 | -4 | 0.041 | 4.35 |  |
| 5 | L Angular gyrus | 39 | -28 | -58 | 44 | 0.049 | 5.20 | 3224 |
|  | L IPL | 40 | -34 | -48 | 44 | 0.042 | 4.52 |  |
| 6 | R STG | 22 | 54 | -32 | 4 | 0.061 | 6.29 | 2768 |
| 7 | R Precentral gyrus | 4 | 54 | -8 | 38 | 0.049 | 5.22 | 2224 |
|  | R Postcentral gyrus | 43 | 62 | -6 | 12 | 0.038 | 4.10 |  |
| 8 | R Culmen (Cerebellum, anterior lobe) |  | 36 | -66 | -26 | 0.054 | 5.65 | 1344 |
| 9 | R STG | 22 | 58 | -10 | -4 | 0.046 | 4.87 | 1248 |
| R thalamus | | | | | | | | |
| 1 | R Medial dorsal nucleus (Thalamus) |  | 12 | -18 | 4 | 0.096 | 10.20 | 19160 |
|  | R Thalamus |  | 12 | -8 | 12 | 0.076 | 8.52 |  |
|  | L Thalamus |  | -8 | -18 | 4 | 0.071 | 8.07 |  |
|  | R Thalamus |  | 22 | -28 | 0 | 0.045 | 5.52 |  |
|  | L Thalamus |  | -6 | -26 | -4 | 0.043 | 5.28 |  |
|  | R Lentiform nucleus (Putamen) |  | 22 | 6 | 4 | 0.038 | 4.81 |  |
|  | R Claustrum |  | 34 | 16 | 2 | 0.028 | 3.64 |  |
| 2 | L IFG | 9 | -40 | 10 | 26 | 0.058 | 6.84 | 7768 |
|  | L Precentral gyrus | 4 | -50 | -4 | 44 | 0.043 | 5.29 |  |
|  | L IFG | 9 | -50 | 16 | 28 | 0.038 | 4.82 |  |
|  | L Precentral gyrus | 6 | -56 | -4 | 24 | 0.032 | 4.08 |  |
|  | L TTG | 42 | -60 | -10 | 14 | 0.032 | 4.02 |  |
|  | L STG | 22 | -58 | -10 | 0 | 0.028 | 3.56 |  |
| 3 | R Insula |  | 34 | 24 | -6 | 0.062 | 7.18 | 3320 |
|  | R Insula | 13 | 42 | 20 | 8 | 0.041 | 5.05 |  |
|  | R IFG | 45 | 52 | 24 | 2 | 0.025 | 3.16 |  |
| 4 | L Fusiform gyrus | 37 | -44 | -58 | -18 | 0.046 | 5.67 | 3112 |
|  | L Fusiform gyrus | 37 | -40 | -66 | -8 | 0.027 | 3.43 |  |
| 5 | R STG | 22 | 56 | -30 | 4 | 0.049 | 5.92 | 2400 |
|  | R STG | 41 | 62 | -16 | 4 | 0.031 | 4.01 |  |
| 6 | L Insula |  | -36 | 20 | -2 | 0.067 | 7.66 | 2000 |
|  | L Insula | 13 | -44 | 20 | -2 | 0.052 | 6.29 |  |
| 7 | L Mammillary body (Thalamus) |  | -10 | -18 | 4 | 0.078 | 8.66 | 1952 |
|  | R Pulvinar (Thalamus) |  | 24 | -30 | 4 | 0.038 | 4.80 |  |
|  | L Thalamus |  | -6 | -30 | -4 | 0.037 | 4.65 |  |
|  | R Pulvinar (Thalamus) |  | 12 | -30 | 12 | 0.034 | 4.33 |  |
|  | R Pulvinar (Thalamus) |  | 6 | -30 | 0 | 0.032 | 4.11 |  |
| 8 | L Claustrum |  | -32 | 20 | -2 | 0.078 | 8.69 | 1584 |
| 9 | L IFG | 45 | -50 | 20 | 0 | 0.044 | 5.38 | 1416 |
|  | L MFG | 46 | -42 | 26 | 20 | 0.037 | 4.63 |  |
| 10 | L Insula | 13 | -46 | 16 | -2 | 0.046 | 5.68 | 1336 |
|  | L Claustrum |  | -34 | 14 | 0 | 0.038 | 4.72 |  |
|  | L Insula |  | -50 | 8 | -2 | 0.036 | 4.54 |  |
| 11 | L Cingulate gyrus | 32 | -4 | 26 | 36 | 0.049 | 6.00 | 1256 |
| 12 | L Cingulate gyrus | 32 | 0 | 20 | 38 | 0.068 | 7.81 | 1120 |

Note: MNI Coordinates correspond to cluster peaks, and anatomical labels indicate gray matter nearest to the cluster peaks. Please refer to the online data repository for cluster analyses with full reports of structures included in each cluster. L: Left, R: Right, FGmed: Medial frontal gyrus, IFG: Inferior frontal gyrus, IPL: Inferior parietal lobule, MFG: Middle frontal gyrus, MOG: Middle Occipital Gyrus, MTG: Middle temporal gyrus, STG: Superior temporal gyrus, TTG: Transverse temporal gyrus.
